# Supplementary material for: Assessing antimicrobial use patterns in Christian Health Association of Malawi (CHAM) health facilities: A cross-sectional study protocol
Source: PLoS One. 2024 Dec 18;19(12):e0306509. doi: 10.1371/journal.pone.0306509 (PMC11654944; doi:10.1371/journal.pone.0306509)
Supplement: S1 File — (PDF) [file pone.0306509.s001.pdf]

| # in original list | Name of facility | District | Randomly generated number |
|---|---|---|---|
| | | | |
| **FINAL SELECTED AND FILTERED** | | | |
| | **CENTRAL REGION** | | |
| 5 | St Cynthia chisankhwa HC | Chitipa | 5 |
| 14 | Ekwendeni Hospital | Mzimba | 14 |
| 17 | Padro Pio Mzimba | Mzimba | 17 |
| 18 | St John's Hospital | Mzimba | 18 |
| 27 | Luwazi HC | Nkhatabay | 27 |
| | | | |
| | **CENTRAL REGION** | | |
| 32 | Mtendere Community Hospital | Dedza | 32 |
| 33 | Mua Hospital | Dedza | 33 |
| 41 | St Joseph Chiphwaya HC | Dedza | 41 |
| 42 | Kaundu HC | Dedza | 42 |
| 44 | Madisi Hospital | Dowa | 44 |
| 46 | Nkhamenya Community Hospital | Kasungu | 46 |
| 78 | Ganya HC | Ntcheu | 78 |
| 85 | Nsipe HC | Ntcheu | 85 |
| 92 | Chinthembwe HC | Ntchisi | 92 |
| 95 | Kaphatenga HC | Salima | 95 |
| | | | |
| | **SOUTHERN REGION** | | |
| 112 | Mindati HC | Chikhwawa | 112 |
| 113 | St Joseph Hospital Nguludi | Chiradzuro | 113 |
| 120 | Nthorowa HC | Machinga | 120 |
| 125 | Katema HC | Mangochi | 125 |
| 137 | Billy Riodan HC | Mangochi | 137 |
| 158 | St Joseph Mitengo | Thyolo | 158 |
| 166 | Chilipa HC | Zomba | 166 |

| **FACLITIES LIST AND SELECTION PROCESS INVOLVING MATCHING NUMBERS** | | | |
|---|---|---|---|
| **#** | **Name of facility** | **District** | **Randomly generated numbers** |
| | | | |
| | **CENTRAL REGION** | | |
| 1 | Kaseye Community Hosp | Chitipa | |
| 2 | Chambo | Chitipa | |
| 3 | Msumbe HC | Chitipa | |
| 4 | St Clara HC | Chitipa | |
| 5 | St Cynthia chisankhwa HC | Chitipa | 5 |
| 6 | St Peters Community Hosp | Likoma | |
| 7 | St Mary's HC (Chizumulu) | Likoma | |
| 8 | Chigwere HC | Mzimba | |
| 9 | Atupele Community Hospital | Karonga | |
| 10 | St Annie's Chilumba HC | Karonga | |
| 11 | Sangiro HC | Karonga | |
| 12 | Katete Community Hospital | Mzimba | |

| | | | |
|---|---|---|---|
| 13 | Mzambazi Community Hospital | Mzimba | |
| 14 | Ekwendeni Hospital | Mzimba | 14 |
| 15 | Embangweni Hospital | Mzimba | |
| 16 | St John of God Mental Hospital- Mz | Mzimba | |
| 17 | Padro Pio Mzimba | Mzimba | 17 |
| 18 | St John's Hospital | Mzimba | 18 |
| 19 | Enukweni HC | Mzimba | |
| 20 | Kalikumbi HC | Mzimba | |
| 21 | Lunjika HC | Mzimba | |
| 22 | Mabiri HC | Mzimba | |
| 23 | Mharaunda HC | Mzimba | |
| 24 | Engalaweni HC | Mzimba | |
| 25 | Chilambwe HC | Nkhatabay | |
| 26 | Liwaladzi HC | Nkhatabay | |
| 27 | Luwazi HC | Nkhatabay | 27 |
| 28 | David Gordon Memorial Hospital | Rumphi | |
| 29 | Luwuchi HC | Rumphi | |
| 30 | Tcharo HC | Rumphi | |
| 31 | Nthenje | Rumphi | |
| | | | |
| | **CENTRAL REGION** | | |
| 32 | Mtendere Community Hospital | Dedza | 32 |
| 33 | Mua Hospital | Dedza | 33 |
| 34 | Kanyama HC | Dedza | |
| 35 | Kasina HC | Dedza | |
| 36 | Matumba HC | Dedza | |
| 37 | Mikondo HC | Dedza | |
| 38 | Mphunzi HC | Dedza | |
| 39 | Nakalanzi HC | Dedza | |
| 40 | St Annes Bembeke | Dedza | |
| 41 | St Joseph Chiphwaya HC | Dedza | 41 |
| 42 | Kaundu HC | Dedza | 42 |
| 43 | St Cornelius | Dedza | |
| 44 | Madisi Hospital | Dowa | 44 |
| 45 | Mvera HC | Dowa | |
| 46 | Nkhamenya Community Hospital | Kasungu | 46 |
| 47 | St Andrews Community Hospital | Kasungu | |
| 48 | Mpasadzi HC | Kasungu | |
| 49 | St Faith Anglican Clinic | Kasungu | |
| 50 | Chilanga HC | Kasungu | |
| 51 | St Denis HC | Kasungu | |
| 52 | ABC Community clinic | Lilongwe | |
| 53 | Mlare Community Hospital | Lilongwe | |
| 54 | Mtengowanthenga Community | Lilongwe | |
| 55 | Nambuma Community Hospital | Lilongwe | |
| 56 | Partners in Hope Community | Lilongwe | |
| 57 | Daeyang Lukes Hospital | Lilongwe | |
| 58 | Likuni Hospital | Lilongwe | |
| 59 | Nkhoma Hospital | Lilongwe | |

| | | | |
|---|---|---|---|
| 60 | St Gabriel’s Hospital | Lilongwe | |
| 61 | St John of God Mental Hospital- LL | Lilongwe | |
| 62 | Dzenza HC | Lilongwe | |
| 63 | Malingunde HC | Lilongwe | |
| 64 | Mbwatalika HC | Lilongwe | |
| 65 | Blessing Community | Lilongwe | |
| 66 | Child Legacy Community | Lilongwe | |
| 67 | Dorcas HC | Lilongwe | |
| 68 | St Chavala | Lilongwe | |
| 69 | Our Lady of Mount Carmel | Mchinji | |
| 70 | St Joseph/Ludzi Rural Hospital | Mchinji | |
| 71 | St Michaels Guilleme | Mchinji | |
| 72 | Alinafe Community Hospital | Nkhotakota | |
| 73 | St Annes Hospital | Nkhotakota | |
| 74 | Kapiri HC | Nkhotakota | |
| 75 | Kasitu HC | Nkhotakota | |
| 76 | Sr. Teleza Community Hospital | Ntcheu | |
| 77 | Chigodi HC | Ntcheu | |
| 78 | Ganya HC | Ntcheu | 78 |
| 79 | Gowa HC | Ntcheu | |
| 80 | Lakeview HC | Ntcheu | |
| 81 | Matanda HC | Ntcheu | |
| 82 | Mlanda HC | Ntcheu | |
| 83 | Muluma HC | Ntcheu | |
| 84 | Nzama | Ntcheu | |
| 85 | Nsipe HC | Ntcheu | 85 |
| 86 | Ntonda HC | Ntcheu | |
| 87 | Phalula HC | Ntcheu | |
| 88 | Senzani HC | Ntcheu | |
| 89 | Sharpevalley HC | Ntcheu | |
| 90 | Tsangano HC | Ntcheu | |
| 91 | Livulezi HC | Ntcheu | |
| 92 | Chinthembwe HC | Ntchisi | 92 |
| 93 | Malambo St Thereza HC | Ntchisi | |
| 94 | Chitala HC | Salima | |
| 95 | Kaphatenga HC | Salima | 95 |
| 96 | Ngodzi HC | Salima | |
| 97 | Senga Bay Baptist HC | Salima | |
| 98 | Thavite HC | Salima | |
| | | | |
| | **SOUTHERN REGION** | | |
| 99 | Comfort Community Hospital | Balaka | |
| 100 | Kankao HC | Balaka | |
| 101 | St Peters/Utale I HC | Balaka | |
| 102 | Ulongwe HC | Balaka | |
| 103 | Utale II HC | Balaka | |
| 104 | Mlambe Hospital | Blantyre | |
| 105 | Chileka SDA HC | Blantyre | |
| 106 | Lumbira HC | Blantyre | |

| | | | |
|---|---|---|---|
| 107 | Malabada HC | Blantyre | |
| 108 | St Vincent HC | Blantyre | |
| 109 | Soche Clinic | Blantyre | |
| 110 | Montfort Hospital | Chikhwawa | |
| 111 | Misomali HC | Chikhwawa | |
| 112 | Mindati HC | Chikhwawa | 112 |
| 113 | St Joseph Hospital Nguludi | Chiradzuro | 113 |
| 114 | PIM HC | Chiradzuro | |
| 115 | Chaone HC | Machinga | |
| 116 | Gawanani HC | Machinga | |
| 117 | Mpiri HC | Machinga | |
| 118 | Namandanje HC | Machinga | |
| 119 | Nsanama HC | Machinga | |
| 120 | Nthorowa HC | Machinga | 120 |
| 121 | Koche Community Hospital | Mangochi | |
| 122 | Mulibwanji Community Hospital | Mangochi | |
| 123 | St Martins Community Hospital | Mangochi | |
| 124 | Kapire HC | Mangochi | |
| 125 | Katema HC | Mangochi | 125 |
| 126 | Lulanga HC | Mangochi | |
| 127 | Makanjira HC | Mangochi | |
| 128 | Malembo HC | Mangochi | |
| 129 | Mase HC | Mangochi | |
| 130 | Mpondasi HC | Mangochi | |
| 131 | Nankhwali HC | Mangochi | |
| 132 | Nkope HC | Mangochi | |
| 133 | Saiti Masungu HC | Mangochi | |
| 134 | Sr Martha HC | Mangochi | |
| 135 | St Marys Community Mangochi | Mangochi | |
| 136 | Mkucinga Dwa Mbone HC | Mangochi | |
| 137 | Billy Riodan HC | Mangochi | 137 |
| 138 | Mulanje Hospital | Mulanje | |
| 139 | Namasalima HC | Mulanje | |
| 140 | Namulenga HC | Mulanje | |
| 141 | Mloza HC | Mulanje | |
| 142 | Matandani HC | Neno | |
| 143 | Matope HC | Neno | |
| 144 | Neno Parish HC | Neno | |
| 145 | Nsambe HC | Neno | |
| 146 | Kalemba Community Hospital | Nsanje | |
| 147 | Trinity Hospital | Nsanje | |
| 148 | Chididi HC(NSJ) | Nsanje | |
| 149 | Lulwe HC | Nsanje | |
| 150 | Holy Family Hospital | Phalombe | |
| 151 | Chiringa HC | Phalombe | |
| 152 | Mwanga HC | Phalombe | |
| 153 | Sukasanje HC | Phalombe | |
| 154 | Chingadzi Community Hospital | Thyolo | |
| 155 | Malamulo Hospital | Thyolo | |

| | | | |
|---|---|---|---|
| 156 | Chipho HC | Thyolo | |
| 157 | Makapwa HC | Thyolo | |
| 158 | St Joseph Mitengo | Thyolo | 158 |
| 159 | St Martins/Molele HC | Thyolo | |
| 160 | Thembe HC | Thyolo | |
| 161 | Thomas HC | Thyolo | |
| 162 | Mbalanguzi | Thyolo | |
| 163 | Chuluchosema HC | Zomba | |
| 164 | Pirimiti Hospital | Zomba | |
| 165 | St Luke's Hospital | Zomba | |
| 166 | Chilipa HC | Zomba | 166 |
| 167 | Chipini HC | Zomba | |
| 168 | H Parker HC | Zomba | |
| 169 | Magomero HC | Zomba | |
| 170 | Matiya HC | Zomba | |
| 171 | Mayaka HC | Zomba | |
| 172 | Namalaka HC | Zomba | |
| 173 | Namikango Maternity Clinic | Zomba | |
| 174 | Nkasala HC | Zomba | |
| 175 | Sitima HC | Zomba | |
