# Supplementary material for: Assessing antimicrobial use patterns in Christian Health Association of Malawi (CHAM) health facilities: A cross-sectional study protocol
Source: PLoS One. 2024 Dec 18;19(12):e0306509. doi: 10.1371/journal.pone.0306509 (PMC11654944; doi:10.1371/journal.pone.0306509)
Supplement: S2 File — (PDF) [file pone.0306509.s002.pdf]

STUDY DATA COLLECTION TOOL	
**FACILITY DATA**	
**Question**	Response
A. DEMOGRAPHIC DATA	
1. Facility Name	Name
2. Facility type (Use Capital letters)	Full hospital, Community Hospital, Health Centre
3. Hospital District (Use Capital letters)	Name
4. Region of the facility	North, Central, East, South
5. Reachability of the facility	No road, Hard to reach, close to main road (a kilometer), tarmac to facility
6. Population of the catchment area	Number
7. Facility In-charge	NurseClinicianSister or nunFather or PriestPharmacy personnelMedical DirectorAdministratorHuman ResourceLab PersonnelOthers
B. MEDICINE USE AT A FACILITY LEVEL	
8. Who prescribes to patients	Clinicians or Physicians (Medical Doctors)NursesPharmacy personnelLab personnelSister (no clinical or nursing background)Others
9. Is Malawi Standard Treatment Guidelines (MSTG) available (computer or phone or physical copy)	Yes or no
10. Does the hospital has its own formulary list adapted from Malawi Essential medicines List?	Yes or no
11. From your Pharmacy list, how may are Medicines,	Number
13. From the medicines in 11, How many are antibiotics?	Number
14. Out of those in 13, how many antibiotics are in stock?	Number
15. What is sum number of days for key antibiotics to be out of stock in a months (add all days of stock out per antibiotic, then add all total)?	Number
16. What is the cost of all antibiotics in stock (from Excel of your order)?	Number
17. What is the cost of all medicines procured in the previous month?	Number
18. Who is heading the pharmacy	PharmacistPharmacy TechnicianPharmacy AssistantNurseClinicianNun or sister (no pharmacy training)Father or priest (no pharmacy training)Lab personnelPharmacy attendantsOthers
19. Does the pharmacy has a pharmacy profession (person who studied pharmacy)	Yes or no
20. Who dispenses medicines at OPD	ClinicianPharmacy personnel (professional)NurseLab personnelNun or sister (no pharmacy training)Pharmacy attendantFather or PriestOthers
21. How long does it take to dispense medicines at OPD on average?	1 minute2 minutes3 minutes4 minutes5 minutes6. minutes7 minutes8 minutes9 minutes10 minutes11-15 minutes16-20 minutes21-25 minutes26-30 minutes
Please collect GPS location for the health facility	

PATIENT AND PRESCRIPTION DATA	
A. DEMOGRAPHIC DATA	
1. Facility name (E.g. DGMH, Sangiro, Child Legacy, Ludzi, Guillime, OLMC, Nguludi, Ekwendeni, Mlambe)	Type
2. Facility Type	Full hospital, Community Hospital, Health Centre
3. Facility Region	North, Central, East, South
4. Facility district (Rumphi, Lilongwe, Ntcheu)	Type

5. Patient ID (number)	Automated
6. Patient age	choose one
7. Patient sex	choose one
B. PATIENT AND PRESCIRPTION DATA	
8. Presenting complaint (choose one or many)	choose one
9. If presenting complaint or symptom not listed in 7 above, type in this box	type
10. Were vital signs collected/ measured	Yes or no
11. Were lab tests requested by a prescriber?	Yes or no
12. Which lab tests were requested? (choose one or many)	many
13. From lab tests, was cultures and sensitity test done?	Yes or no
14. For cultures and sensitity test, were specimen collected?	Yes or no
15. Which lab are cultures done or sent to? (choose one or many)	Name
16. Diagnosis or impression (in passport or patient file) (choose one or many)	type
17. If the diagnosis is not above OR If cancer (specify) type using simple words	type
18. Did the patient undergo caesarian section?	Yes or no
19. Who prescribed the medicines (know or ask patient)	many
20. On avarage, how long does it take for you to dispense Medicines to one patients or How long did it take for medicines to be dispensed for the patient interviewed?	Time
21. Who dispensed medicines	choose one
22. Did you receive all antibiotics prescribed	Yes, some or no
23a. If no in 22, Which antibiotic not received after prescribing 1 (WRITE FIRST ANTIBIOTIC BY NAME)	type
23b. If no in 22, Which antibiotic not received after prescribing 2 (WRITE FIRST ANTIBIOTIC BY NAME)	type
23c. If no in 22, Which antibiotic not received after prescribing 3 (WRITE FIRST ANTIBIOTIC BY NAME)	type
23d. If no in 22, Which antibiotic not received after prescribing 4 (WRITE FIRST ANTIBIOTIC BY NAME)	type
23e. If no in 22, Which antibiotic not received after prescribing 5 (WRITE FIRST ANTIBIOTIC BY NAME)	type
24. Do you know how to take the medicines	Yes or no
25. Are the Medicines given in pill pack or Container or original proper package?	Yes or no
26. If given in pill pack or Container or original package, is it properly labelled? (Drug name, Total number of meds packed, dosing and expiry	Yes or no
27. Patient's Number of hospital visit in the last 30 days	Number
**28. Prescribed Medicines **	many
**Medicine Name (Generic Name)**	Yes or no
**Strength**	choose one
**Dose**	choose one
**Route**	choose one
**Frequency**	choose one
**Duration**	choose one
**Generic name?**	choose one
